# Air pollution and DNA methylation: effects of exposure in humans

**DOI:** 10.1186/s13148-019-0713-2

**Published:** 2019-09-03

**Authors:** Christopher F. Rider, Chris Carlsten

**Affiliations:** 1 Respiratory Medicine, Faculty of Medicine, Chan-Yeung Centre for Occupational and Environmental Respiratory Disease (COERD), University of British Columbia, Vancouver, British Columbia Canada; 2 Diamond Health Care Centre 7252, 2775 Laurel Street, Vancouver, BC V5Z 1 M9 Canada; 3 Institute for Heart and Lung Health, University of British Columbia, Vancouver, British Columbia Canada; 4 School of Population and Public Health, University of British Columbia, Vancouver, British Columbia Canada

**Keywords:** Epigenetics, 5-Methylcytosine, Epidemiology, Controlled human exposure studies, Diesel exhaust

## Abstract

Air pollution exposure is estimated to contribute to approximately seven million early deaths every year worldwide and more than 3% of disability-adjusted life years lost. Air pollution has numerous harmful effects on health and contributes to the development and morbidity of cardiovascular disease, metabolic disorders, and a number of lung pathologies, including asthma and chronic obstructive pulmonary disease (COPD). Emerging data indicate that air pollution exposure modulates the epigenetic mark, DNA methylation (DNAm), and that these changes might in turn influence inflammation, disease development, and exacerbation risk. Several traffic-related air pollution (TRAP) components, including particulate matter (PM), black carbon (BC), ozone (O_3_), nitrogen oxides (NO_*x*_), and polyaromatic hydrocarbons (PAHs), have been associated with changes in DNAm; typically lowering DNAm after exposure. Effects of air pollution on DNAm have been observed across the human lifespan, but it is not yet clear whether early life developmental sensitivity or the accumulation of exposures have the most significant effects on health. Air pollution exposure-associated DNAm patterns are often correlated with long-term negative respiratory health outcomes, including the development of lung diseases, a focus in this review. Recently, interventions such as exercise and B vitamins have been proposed to reduce the impact of air pollution on DNAm and health. Ultimately, improved knowledge of how exposure-induced change in DNAm impacts health, both acutely and chronically, may enable preventative and remedial strategies to reduce morbidity in polluted environments.

**Electronic supplementary material:**

The online version of this article (10.1186/s13148-019-0713-2) contains supplementary material, which is available to authorized users.

## Introduction

Air pollution is well known to be harmful to health, and emerging data support the hypothesis that exposure to air pollution may contribute to the development of lung conditions, metabolic disorders, and cardiovascular disease [1]. While there are acute effects of exposure to air pollutants, we do not understand if exposures during gestation or childhood have a greater impact on disease development than those experienced as an adult, or if morbidity is simply driven by the accumulation of exposures. In addition to health effects, exposure to air pollution can alter epigenetic marks, in particular, DNA methylation (DNAm). However, our understanding of how air pollution modulates DNAm in the lungs and beyond, and how these changes in DNAm influence the associated health outcomes, remains modest. Studies suggest that air pollution exposure often results in a widespread decrease in DNAm, but we do not understand whether such effects are targeted to specific sites or scattered across the genome globally due to untargeted effects on epigenetic control mechanisms. Here, we review the in vivo effects of air pollution exposure on DNAm and briefly discuss the association of DNAm with lung health in humans.

### Air pollution

Air pollution is a complex mixture of particulate matter and gasses that are produced by multiple industrial, commercial, and individual activities [2–4]. Traffic-related air pollution (TRAP) is a significant source in urban environments, especially of particulate matter (PM), which includes black carbon (BC), absorbed metals, and polyaromatic hydrocarbons (PAHs) of various size fractions, the smallest of which can penetrate deep into the lungs [3, 5]. TRAP also includes gasses, such as nitrogen oxides (e.g., NO_2_, NO_*x*_) and sulfur dioxide (SO_2_). The interaction of NO_2_ and PAHs with heat and sunlight results in the formation of highly reactive ground-level ozone (O_3_) [6]. Individual components of air pollution mixtures are rarely encountered in isolation within natural settings, which makes attribution to particular elements challenging outside of single-source or component controlled human exposure studies [7, 8].

Owing to its complex composition, the mechanisms involved in the health effects of air pollution are not entirely clear and could include direct oxidative effects of O_3_ or induction of reactive oxygen species (ROS) following PM exposure. The oxidative stress induced by exposures mediates activation of downstream inflammatory pathways, such as mitogen-activated protein kinase (MAPK), nuclear factor-kappa B (NF-κB) and activator protein 1 (AP1), leading to increased cytokine expression, activation of immune cells, and ultimately inflammation. Epigenetic mechanisms may also contribute to the development and maintenance of inflammation and conditions such as asthma [9–11]. Epigenetic mechanisms include changes in histone tail modifications, miRNA expression, and DNAm.

### DNA methylation

DNAm describes the attachment of methyl groups to DNA, usually at the fifth carbon of cytosines, leading to the formation of 5-methylcytosine (5-mC) [12]. In mammals, DNAm predominantly occurs at C-G dinucleotides, referred to as CpGs. Only approximately 1% of bases and 5% of cytosines across the genome are methylated, but 60–80% of CpGs are methylated in individual human somatic cells [13]. Though controversial, DNAm in the promoter regions of genes may contribute, along with histone variants, histone modifications, and non-coding RNAs, to the regulation of gene expression [11, 12]. CpGs are sparsely and non-randomly distributed in much of the genome, but gene promoter regions often contain “CpG islands” which consist of areas of approximately 1 kb enriched with CpG sites and flanked on either side by regions known as “CpG shores” and then by “CpG shelves” [14]. Un- or sparsely methylated promoter CpG islands are correlated with active gene expression and are often located near constitutively expressed housekeeping genes, while methylated CpG island promoters may be associated with reduced expression of the proximal gene [12, 14, 15]. DNAm of CpG sites located in gene bodies may be related to transcription initiation, elongation efficiency, and alternative splicing [16, 17].

### DNA methylation and demethylation

**Fig. 1:**
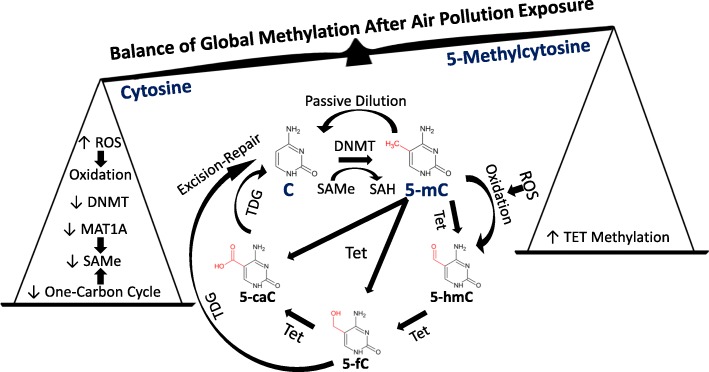
Air pollution-associated effects that may modulate global DNA methylation. Cytosines (C) in CpG sites may be methylated to 5-methylcytosine (5-mC). Ten-eleven translocation methylcytosine dioxygenase (*TET*) family members can catalyze DNA demethylation through converting 5-mC to 5-hydroxymethylcytosine (5-hmC), 5-formylcytosine (5-fC), and 5-carboxycyotosine (5-caC). G/T mismatch-specific thymine-DNA glycosylase (*TDG*) may as part of mismatch excision repair processes excise 5-fC or 5-caC and restore a C. Alternatively, 5-mC may undergo passive dilution and revert to C during mitosis. Numerous factors could affect the balance of cytosine (C) and 5-methylcytosine (5-mC) at CpGs throughout the genome following air pollution exposure. Air pollution-induced reactive oxygen species (ROS) may increase oxidation of 5-mC to 5-hydroxymethylcytosine (5-hmC). Global generation of 5-mC may also be decreased by air pollution-induced reductions in DNA methyltransferase (*DNMT*) expression. Additionally, expression of methionine adenosyltransferase 1A (*MAT1A*) and activity of the one-carbon cycle may be lower, leading to reduced production of the methyl donor S-adenosyl methionine (SAMe) and subsequently 5-mC. Conversely, *TET* DNA methylation may reduce expression and subsequently decrease TET activity, which could contribute to maintaining 5-mC

DNAm is driven and maintained by the activity of DNA methyltransferases (DNMT) (Fig. 1) [16, 18]. The methyl groups needed are transferred from S-adenosyl methionine (SAMe), which is generated by members of the methionine adenosyltransferase (MAT) enzyme family as part of the one-carbon cycle [12, 16]. DNA demethylation occurs passively through a lack of maintenance during cell division or by the activity of enzymes, including ten-eleven translocation methylcytosine dioxygenase (TET) family members. TETs convert 5-mC to 5-hydroxymethylcytosine (5-hmC) and then to 5-formylcytosine (5-fC) and finally 5-carboxycyotosine (5-caC), along with other related conversions (Fig. 1) [12, 13, 16]. Finally, G/T mismatch-specific thymine-DNA glycosylase (*TDG*) excises 5-fC or 5-caC and restores an unmethylated cytosine through excision repair [13, 19]. 5-mC can also be deaminated to uracil, which can be restored to cytosine again via excision repair [19].

### Measuring DNA methylation

DNAm is often assessed through bisulphite conversion of DNA, though other techniques use high-performance liquid chromatography, mass spectrometry, antibodies or proteins that bind methylated DNA, or methylation-sensitive restriction enzymes [20, 21]. With bisulphite conversion, 5-hmC cannot be distinguished from 5-mC, but other techniques are capable of this distinction [20, 22]. The DNA bases 5-mC and 5-hmC have been shown to have different effects on gene transcription, and it may, therefore, be important to be able to differentiate these bases to further understand the relative impact they have on disease [23].

PCR and pyrosequencing techniques allow individual sites to be assessed with high accuracy and can be used to analyze specific gene regions or repetitive elements, including long interspersed nuclear element (LINE) 1 and Alu repeats, which are used (albeit imperfectly) as surrogates for global DNAm [12, 24]. Array-based methods, particularly the Illumina Infinium HumanMethylation 450K (~ 450,000 CpGs) and HumanMethylationEPIC platform (~ 850,000 CpGs), have dramatically increased the efficiency of throughput but, due to decreasing cost, next-generation bisulfite sequencing is becoming common, even though it poses its own analysis challenges [25, 26].

### Epidemiological, population, and controlled crossover study designs

The effects of air pollution on DNAm have been investigated using several different study designs [27].

#### Epidemiological-land use regression study design

Many studies use an epidemiological study approach in which concentrations of specific air pollutants are estimated, using factors such as geography, traffic patterns, and fixed air pollution sensor data, to generate land use regression (LUR) models [28]. Volunteers are recruited and give samples (most often blood), and average air pollutant concentrations at each volunteer’s home address are estimated using LUR models, over various time windows (usually 1, 3, 7, and 30 days) before their sample is collected [29–31]. Estimated average air pollution concentrations across multiple volunteers for each different time window are then correlated with data on global DNAm or methylation at specific CpG sites, with significance calculated from the fit of such correlations, and models adjusted for confounding factors. The health effects of exposures can then be investigated, provided that air pollution concentrations vary over time [5, 28, 32]. This approach has the benefits of allowing real-world exposures to be studied but has several limitations, including difficulties in controlling for personal factors among volunteers, choosing the appropriate time window, and often not having a large concentration range within the study period [27, 28, 31, 32].

#### Population study design

An alternative to an epidemiological study approach is to compare people living in areas with different air pollution concentrations, for example, an area with lots of industry and high air pollution with a rural area nearby with low air pollution [27, 32–34]. After correction for differences between the populations living in these two locations, comparison allows estimation of the effects of the increased air pollution exposure associated with living in the industrial area. This study design has limitations, principally in matching characteristics of the volunteers living in the different regions, as there are, for example, often substantial wealth, education, and health care access disparities between areas with high and low air pollution [27, 32, 33, 35].

#### Controlled crossover exposure studies

In controlled crossover exposure studies, volunteers attend a facility where they are exposed to air pollution of a set concentration for a set time [27, 36, 37]. After a suitable washout period, volunteers return to the facility and receive an equivalent control exposure. By comparing the response to the control and air pollution exposure in each individual, across all the volunteers, the effects of the exposure can be accurately assessed. As each individual serves as their own control, the confounding effects of factors such as age and ethnicity are reduced [36, 38, 39]. However, such studies are difficult to implement, have considerable costs in terms of running facilities, and may be susceptible to carryover effects if washout periods are not sufficiently long. In summary, each of these experimental approaches has strengths and weaknesses, and there are therefore benefits to conducting multiple experiments using different methodologies to determine the effects of air pollution on DNAm.

## Effects of air pollutants on DNA methylation across the life course

DNAm data from different studies is measurable using standardized platforms and is reflective of transcription factor binding and gene expression. DNAm is, therefore, a logical tool for trying to understand air pollution’s effect on genomic function and downstream measures of interest to health. Here, we outline research examining interactions between DNAm and air pollution, using a life stage approach [40].

### Effects of air pollution exposure in utero

To understand whether in utero air pollution exposure affects DNAm, a number of studies have examined placenta or cord blood samples [33, 34, 41, 42]. One study showed a significant concentration-dependent association between exposure to NO_2_ during pregnancy and DNAm at three CpGs in cord blood samples measured using the Illumina 450K platform (Table 1 and Additional file 2: Table S2) [41]. The authors separately evaluated 739 CpGs associated with 38 antioxidant and anti-inflammatory genes, identifying two differentially methylated genes, thyroid peroxidase (*TPO*) and catalase, which may have roles in responses to ROS. The association of NO_2_ exposure with *SLC25A28* and *TPO* DNAm was validated in blood samples from cohorts of 4- and 8-year-old children [41]. NO_2_ is typically viewed as a surrogate for road traffic in outdoor city settings but is not necessarily independent of other TRAP-related exposures.

**Table 1:** Detail on the studies reviewed. This table describes features of the studies described in this review, including the first author’s surname, study location, detail on cohorts, volunteer number, sample types collected, and study details. *NA* not applicable, *ND* not determined, *NR* not reported, *LINE* long interspersed nuclear element, *LUR* land use regression. For additional details on the studies reviewed, please see Additional file 1: Table S1

Study Author - Year	Cohorts	Study Location(s)	Number of Volunteers	Average Age (years)	Sample Type(s)	DNAm Analysis	Air Pollution Analysis
**In Utero Studies**							
Gruzieva 2017	MeDALL	Europe	280	0	Cord Blood	Illumina 450 K	ESCAPE project LUR NO_2_ data
	Generation R	Netherlands	809	0	Cord Blood		
	CHS	USA	226	0	Cord Blood		
	MoBa	Norway	193	0	Cord Blood		
	BAMSE	Sweden	733	4.4	Blood		
	MeDALL	Europe	444	8.2	Blood		
	BAMSE EpiGene	Sweden	342	8.3	Blood		
Goodrich 2016	Mother and Child Environmental	Durban, South Africa	22	26.1	Cord Blood	Illumina 450 K	NO_x_ exposures from LUR
Maghbooli 2018	Low pollution area	Tehran, Iran	44	30.09	Placenta	HPLC	High and Low Pollution Areas
	High pollution area		48	30			
Cai 2017	Normal	Wenzhou, China	101	26.7	Placenta	Pyrosequencing	PM_10_ from LUR models
	Fetal Growth Restriction	Wenzhou, China	80	26.7			
Kingsley 2016	Rhode Island Child Health Study	Providence, USA	471	30	Placenta	Illumina 450 K and pyrosequencing	Mothers categorized by distance from highways
**Studies in Children**							
Breton 2016	Children’s Health Study	California, USA	459	11.2	Blood	Pyrosequencing	LUR for PM_2.5_, PM_10_, NO_2_ and O_3_
Hew 2015	NA	Fresno, USA	171	13.7	Blood T regulatory cells	Pyrosequencing	LUR for PAH
			85	15.1			
Somineni 2016	Exposure Sibling Study for discovery	Cincinnati, USA	70	11	Nasal Brushings (validation in saliva, PBMCs and bronchial epithelial cells)	Illumina 450 K and pyrosequencing	LUR for black carbon
	Pediatric Environmental Exposure Study for replication	Cincinnati, USA	186	12			
Lovinsky-Desir 2017	Non-Active	New York, USA	58	12.7	Buccal swabs (94% squamous epithelial cells)	Pyrosequencing	BC assessed by vest mounted monitors and activity by accelerometer
	Active		77	12.2			
**Studies in Adults**							
Jiang 2014	NA	Vancouver, Canada	16	56	Blood	Illumina 450 K	Controlled exposures to diesel exhaust and filtered air
Bind 2014	Normative Aging Study (NAS)	Boston, USA	777	72	Blood	Pyrosequencing	PM_2.5_, particle number and BC assessed by LUR
Bind 2015	Normative Aging Study (NAS)	Boston, USA	777	72	Blood	Pyrosequencing	PM_2.5_, particle number and BC from LUR
Lee 2017	Normative Aging Study (NAS)	Boston, USA	92	ND	Blood	Probably pyrosequencing	BC and sulfate from LUR
Chi 2016	Multi-Ethnic Study of Atherosclerosis	Baltimore, New York, St. Paul, Winston-Salem USA	1207	69.6	PBMC	Illumina 450 K	PM_2.5_ and NO_x_ concentrations from spatiotemporal models
Panni 2016	KORA F3	Augsburg, Germany	500	53	Blood	Illumina 450 K	PM_2.5_ exposures from LUR models
	KORA F4		1799	61			
	Normative Aging Study	Boston, USA	657	72			
Mostafavi 2017	EPIC Italy Cohort	Italy	95	~ 50	PBMC	Agilent 4x44K and Ilumina 450 K	NO_x_ estimated with LUR
	Northern Swedish Health and Disease Study Cohort	Northern Sweden	455	~ 50	PBMC		
Plusquin 2017	EPIC-Italy	Italy	454	54.2	Blood	Illumina 450 K	PM_10_, PM_2.5_, NO_2_ and NO_x_ from LUR models
	EPIC-Netherlands	Netherlands	159	58.8			
De F.C. Lichtenfels 2018	LifeLines	Groningen, Netherlands	1017	47.3	Blood	Illumina 450 K	NO_2_, PM_10_, PM_2.5_ from LUR models
Mostafavi 2018	EXPOsOMICS	Italy, Netherlands, Switzerland, United Kingdom	157	61	Blood	Illumina 450 K	PM2.5 assessed using backpack sensor, outdoor sensor and LUR model
Lepeule 2014	Normative Aging Study (NAS)	Boston, USA	776	72	Blood	Pyrosequencing	BC, CO, NO_2_, O_3_ and PM_2.5_ were modeled using LUR
Peng 2016	Normative Aging Study	Boston, USA	551	~ 76	Blood	Pyrosequencing	PM2.5 assessed using a hybrid LUR and satelite based model
Zhong 2017	NA	Toronto, Canada	10	19–49	CD4+ Th Cells (~ 96% pure)	Illumina 450 K	Controlled exposures to filtered air and concentrated ambient PM_2.5_
De Nys 2018	NA	Leuven, Belgium	23	22.3	Buccal swabs	UPLC - mass spectrometry	PM_2.5_ and PM_10_ from LUR
Clifford 2017	NA	Vancouver, Canada	16	28.8	Bronchial brushings (~97% epithelial cells)	Illumina 450 K	Controlled exposures to diesel exhaust and allergen, with filtered air and saline controls

In a similar study in cord blood investigating exposure to NO_*x*_, none of the CpGs on the Illumina 450K platform exceeded the cutoff, potentially reflecting the small sample size of the study [34]. Nevertheless, the CpGs with the smallest *p* values in association with NO_*x*_ exposure were enriched within CpG islands, generally hypomethylated and included reduced DNAm of selenoprotein K, an extracellular antioxidant that may contribute to exposure detoxification. These studies indicate that in utero exposure can significantly modulate DNAm, but that differences may be modest when changes in air pollution concentration are small, and therefore results may not pass correction for multiple testing [43]. Additionally, these results suggest that genes associated with the significantly modulated CpG sites may be related to oxidative stress pathways.

In addition to the gasses NO_2_ and NO_*x*_, in utero exposure to PM has also been shown to modulate DNAm. One of the most recent studies recruited 100 expectant mothers, 50 of whom lived in an area of elevated pollution (PM_10_ 80-111 μg/m^3^, PM_2.5_ 35-44 μg/m^3^) and 50 in a region with lower air pollution (PM_10_ 50–62 μg/m^3^, PM_2.5_ 21–30 μg/m^3^) (Table 1; Additional file 1: Table S1) [33]. There were significant changes in mean DNAm in placenta samples that positively correlated with PM_2.5_ and PM_10_ exposure during the first trimester of pregnancy. These effects were surprising given that pollution levels appeared lowest on average (probably due to seasonal variation) during the first trimester of the study, indicating a potential period of enhanced vulnerability during early development. The existence of a period of early vulnerability is supported by another study in placental samples collected from 181 volunteers in China [44]. Higher PM_10_ exposure during the first trimester, as assessed using LUR models developed from four fixed location sensors, was associated with lower LINE1 methylation. Indeed, each 10 μg/m^3^ increase in PM_10_ exposure, relative to the average of ~ 64 μg/m^3^, was associated with a 1.78% decrease in LINE1 methylation. These results demonstrate that air pollution exposure in utero significantly modulates DNAm in a concentration-dependent manner and that the effects of the exposures remain detectable up to 6 months later in placental and cord blood samples collected at birth.

#### Air pollution, DNA methylation, and growth restriction

One common health outcome observed with prenatal exposure to air pollution is intrauterine growth restriction (IUGR), and this may be associated with higher air pollution sensitivity. In a study with case-control comparisons, more significant effects of air pollution on DNAm were found in babies with IUGR than those with healthy growth [44]. Another study showed that mothers living within 150 m of major roads, which had higher air pollution exposure, had babies with lower birth weight, along with lower placental LINE1 methylation and significant changes in DNAm at seven CpGs, compared to mothers living further from major roads (Additional file 2: Table S2) [42]. Although Maghbooli et al. showed higher mean DNAm after air pollution exposure, both Cai et al. and Kingsley et al. suggested that air pollution exposure reduced LINE1 methylation [33, 42, 44]. While the reasons behind this difference are unclear, relevant factors may include the differences between the study populations in China, the USA, and Iran, and between IUGR and normal babies, the use of pyrosequencing versus high-performance liquid chromatography analysis, the air pollution concentrations, and likely pollutant composition. As noted earlier, while LINE1 is often presented as a surrogate for global DNAm, it does so imprecisely relative to high-performance liquid chromatography analysis [24]. These results are relevant given the known associations between air pollution exposure and poor pregnancy outcomes, such as preterm birth [45].

Several possible mechanisms exist whereby DNAm might connect air pollution exposure to birth outcomes. In one study, placental hydroxysteroid 11-beta dehydrogenase 2 (HSD11B2) promoter methylation was higher by 1.03% and 2.23% in association with each 10 μg/m^3^ increase in PM_10_ exposure during the first and second trimester, respectively [44]. HSD11B2 metabolizes glucocorticoids to protect the fetus from higher concentrations of maternal cortisol that may be induced by air pollution exposure [46]. Enhanced HSD11B2 promoter methylation may result in decreased HSD11B2 expression and elevated fetal cortisol exposure, potentially leading to reduced growth, as has been demonstrated for corticosteroid usage during childhood [44, 47].

Air pollution exposure could also decrease fetal growth through other mechanisms, such as through increasing maternal systolic blood pressure, as hypertension during pregnancy has been associated with lower placental blood flow and reduced nutrition delivery [48]. Living in an area with greater air pollution was associated with higher mean placental DNAm and increased systolic blood pressure that almost reached significance, relative to women with a slightly lower exposure (*p* = 0.07 with *n* = 50) [33]. However, the relationship between the different mechanisms is unclear, and none of the DNAm sites that were significantly changed were replicated between the studies by Cai et al. and Maghbooli et al. (or indeed between any of the studies described in this review) (Additional file 2: Table S2). Additional larger studies are therefore needed to shed light on the effects of specific air pollutants on DNAm and the relationship of DNAm changes with birth outcomes.

### Effects of air pollution on DNA methylation in children and adolescents

The connections between air pollution, DNAm, and health are not limited to prenatal exposures and birth outcomes, but effects may persist into childhood. As an example of this, maternal NO_2_ exposure during the third trimester of pregnancy was associated with higher systolic blood pressure in children assessed at the age of 11 [49]. However, no relationship with blood LINE1 DNAm was identified with NO_2_ exposure. But exposure to PM_10_ or O_3_ during the first trimester was associated with lower LINE1 DNAm at birth, while O_3_ exposure during the third trimester was conversely associated with higher LINE1 DNAm [49]. These results highlight the effects of early life exposures and possible differences based upon both the type of pollutant and developmental stage at exposure. Such findings also suggest that the changes induced by air pollution exposure during pregnancy can persist well into childhood. A possible mechanism for the maintenance of the effects of exposure during pregnancy into childhood could be sustained changes in DNAm. However, other factors, such as genotype, may also contribute to shaping outcomes and, in some cases, may also affect DNAm. For example, O_3_ exposure during the first trimester in one study was associated with increased systolic blood pressure only in 11-year-old children with particular DNMT1 or DNMT3B isoforms [49]. This result suggests that variants that affect essential DNAm control genes have the potential to shape responses and health effects of environmental exposures (Fig. 1). These findings also suggest that a possible mechanism underlying modulation of DNAm following exposures could be changes in the expression of key enzymes that regulate DNAm (Fig. 1).

#### Effects of air pollution on TET expression

A recent study examining the impact of air pollution on other DNAm machinery found that black carbon exposure was associated with significantly higher DNAm in nasal brushings at cg23602092 in the promoter region of *TET1* (Table 1 and Additional file 2: Table S2) [50]. There was also a significant association between asthma status and DNAm at this site, with lower mean methylation in asthmatic children that was also replicated in saliva and peripheral blood mononuclear cell (PBMC) samples from children in the Pediatric Environmental Exposure Study [50]. TET1 catalyzes the conversion of 5-mC to 5-hmC. Higher 5-hmC levels in saliva were found in asthmatic children in comparison to non-asthmatic siblings (who had higher *TET1* DNAm and would, therefore, be expected to have lower *TET1* gene expression). In in vitro experiments, exposure of human bronchial epithelial cells to diesel exhaust particulate resulted in lower *TET1* expression at 4 h and higher *TET1* DNAm at 24 h, along with significantly reduced 5-hmC levels [50]. These results suggest that the oxidative stress associated with air pollution exposure results in time-dependent modulation of *TET1* expression, which then affects 5-hmC levels.

#### Air pollution and FOXP3

A few studies have examined the effects of air pollution exposure on DNAm in association with another specific gene, forkhead box P3 (*FOXP3*) [51, 52]. FOXP3 controls the differentiation and activity of T regulatory cells and may, therefore, have a role in diseases such as asthma [51–53]. For example, one group isolated DNA from T regulatory cells in the blood of adolescents living in Fresno, California [51]. They found that *FOXP3* DNAm was significantly higher in association with higher average PAH concentrations over 1 month, 3 months, and 1 year in asthmatics, while in non-asthmatics, higher *FOXP3* DNAm was only seen with average PAH concentrations over 3 months and a year. *FOXP3* DNAm was found to be inversely correlated with FOXP3 protein expression. There were also significant positive associations between PAH exposure and total IgE levels at all time points in non-asthmatics and all except 3 months in asthmatics. The effects of PAH exposure on *FOXP3* DNAm and IgE were maintained in 19 volunteers who were retested 8 months after their initial visit [51].

Similar results were obtained in a study in New York City using accelerometers and personal backpack monitoring to assess the relationship between exercise, black carbon exposure, and *FOXP3* DNAm in cheek swabs [52]. Analysis of *FOXP3* CpGs indicated that among children with high black carbon exposure (> 1.21 μg/m^3^), non-active children (< 1 h of moderate-to-vigorous activity a day) had the highest DNAm at *FOXP3*, while those who were active had ~ 2.5% lower average methylation at specific *FOXP3* sites. There was no association of physical activity with DNAm levels among children with little black carbon exposure. These results suggest that, despite potentially greater black carbon exposure due to the increased breathing associated with activity, exercise may protect against the harms of air pollution exposure on health, in part by modulating DNAm. Indeed, results also indicated a negative correlation between *FOXP3* DNAm at specific CpG sites and the ratio of forced expiratory volume in 1 s to forced vital capacity (FEV_1_/FVC) and forced expiratory flow at 25–75% of pulmonary volume (FEF_25–75%_), suggesting an association with lung function [52]. However, there was no correlation between *FOXP3* promoter DNAm, activity or lung function, and *FOXP3* mRNA expression in cheek cells. These studies suggest that air pollution exposure may induce FOXP3 methylation, which in turn may reduce FOXP3 expression (potentially reducing T regulatory cell function), ultimately promoting asthma morbidity.

The studies described above both concluded that air pollution affects *FOXP3* DNAm, but the timeframes of exposure effects were different, with Hew et al. showing effects only when exposures were averaged over months while Lovinsky-Desir et al. showed changes over 6 days [51, 52]. This discrepancy may reflect differences between the cell types evaluated, the effects of black carbon and PAHs (or other pollutants with which they are correlated), or alternative factors, such as the increased precision of a personal backpack rather than LUR-based pollution assessment. Nevertheless, these studies provide an example of how air pollution exposure could be linked to the development of diseases, such as asthma, through modulation of DNAm at key sites [54].

### Effects of air pollution exposure on DNA methylation in adults

The impact of air pollution on DNAm has also been investigated in adults. It should be noted, however, that in many of these studies, early life exposures were not recorded and may influence the results seen in adult volunteers. In one study from our group, exposure to freshly generated diesel exhaust (DE) (standardized to 300 μg/m^3^ of PM_2.5_) for 2 h modulated DNAm at 2827 CpG sites in blood samples collected 30 h later, relative to filtered air exposure (Table 1) [55]. DE exposure induced a decrease in DNAm on average across the CpGs measured on Illumina 450K chips, with substantial demethylation of promoter regions of genes in the MAPK and NF-κB pathways. This finding suggests that DNAm changes may increase cytokine concentrations by reducing DNAm-mediated repression of inflammatory gene expression. While the link herein is speculative, increased cytokine levels are indeed often seen following controlled exposures [56].

Lower DNAm was also found in a study of older male volunteers living in Boston, which assessed the effects of exposure to PM, black carbon, and O_3_ on DNAm in the blood associated with five immune-related genes, including interleukin (*IL*) 6, coagulation factor III tissue factor (*F3*), interferon gamma (*IFNG*), and intercellular adhesion molecule (*ICAM*) 1 [57]. There were significant associations between the particle number in the first week or black carbon exposure in the third to fourth weeks, before blood draws with DNAm at *F3*. An interquartile increase in particle numbers (~ 15,000 particles per cm^3^) was associated with an 18% decrease in *F3* DNAm, although the apparent lack of cell-type correction in this study raises concern for confounding. Likewise, increased exposure to O_3_ in the 2- to 4-week period before clinic visits was associated with lower DNAm in the promoter region of *ICAM1*. A 1% decrease in *ICAM1* promoter DNAm was correlated with a 0.7% increase in blood ICAM1 protein expression. These results show that air pollution exposure decreases DNAm at specific immune system-related sites and that this is associated with modulation of associated gene expression.

In a follow-up paper, Bind et al. reanalyzed their data on air pollution and DNAm, using an approach where methylation values were separated into ten quantiles according to the degree of pre-existing DNAm for each volunteer and correlation with air pollution assessed for each of these quantiles, rather than with mean DNAm across volunteers [58]. They found differences compared to the earlier analysis, including stronger negative associations between *F3* DNAm and particle number in volunteers, with greater methylation at *F3* (higher deciles) but with *IFNG* at lower deciles. There was a positive association between black carbon exposure and *ICAM1* DNAm at the 90th decile of *ICAM1* DNAm but negative associations with the 10th to 60th deciles. Such an approach may enhance the ability to robustly detect nuances in the effects of air pollution on DNAm.

In addition to quantile analysis, other advanced statistical methods may improve data quality and provide new insights into the relationship between air pollution and DNAm. As an example, a novel multivariate Bayesian variable selection approach was implemented on an analysis of blood DNAm data from 92 volunteers [59]. In comparison with a conventional Bayesian variable selection approach, which identified DNAm of HLA class II histocompatibility antigen, DR alpha chain (*HLA-DRA*), and *IL9* as being associated with mean concentrations of black carbon for the month before each blood draw, the new approach had improved sensitivity and identified *HLA-DRA*, Fc fragment of IgE receptor Ig, and *IL9* in association with black carbon and *IL5* and *CCL11* with sulfate concentrations. Advances in the analysis may lead to greater consistency in the results obtained from studies of air pollution and DNAm, given that, as indicated in Additional file 2: Table S2, there is no consistency among the top CpGs identified in the studies reviewed.

#### Effects of air pollution exposure averaging time and concentration

Studies have shown that averaging air pollution measures over longer time periods often results in stronger associations with DNAm changes [30, 31, 60]. For example, one study examined PM_2.5_ and NO_*x*_ exposures, averaged over a full-year proceeding of blood draws, and looked at the association with DNAm in CD14^+^-purified monocytes from 1264 volunteers [60]. Measurement identified five CpGs (four higher, one lower) that were associated with greater PM_2.5_ exposure (Additional file 2: Table S2). Another study combining several large cohorts investigating the effects of PM_2.5_ exposure on blood DNAm over different periods up to 28 days also showed greater effects over a longer time window of exposure [31]. Two CpGs were positively correlated with 2- and 7-day average PM_2.5_ concentrations, respectively, across the three cohorts (Additional file 2: Table S2). But with a 28-day window, 10 CpGs showed changes in DNAm, three of which had lower DNAm with increasing PM_2.5_ levels, while seven showed higher methylation [31].

The effects of PM_2.5_ on decreasing ICAM1-associated DNAm also seemed to increase over 1-, 7-, and 28-day windows in another study, suggesting that the underlying changes were slow to develop or required air pollution concentration peaks that only occur intermittently over a longer period [61]. However, the impact of PM_2.5_ on ICAM1 DNAm was not maintained at second study visits a few years later, suggesting (as previously shown at an even shorter interval, but less specifically [55]) that the effects of short-term air pollution exposure may not persist over multiple years [61]. These results suggest that air pollution effects on DNAm may be most visible over medium to long time windows. This time-dependent effect indicates that exposures may take some time to modulate DNAm and that DNAm changes may be maintained for extended periods or alternatively highlights the benefits of prolonged air pollution averaging periods in smoothing out concentration spikes that may lead to inconsistent results.

Air pollutant concentration, like time, may also influence the strength of the association with DNAm in adults. One study evaluated the association of NO_*x*_ exposure with PBMC DNAm in a Swedish cohort with low NO_*x*_ levels (7 μg/m^3^) and an Italian cohort with higher average air pollution (NO_*x*_ 94 μg/m^3^) (Additional file 1: Table S1) [62]. Two CpGs on the Illumina 450K platform were associated with long-term NO_*x*_ exposure in the higher pollution cohort, but no significant effects were found in the lower pollution cohort (Additional file 2: Table S2). Similar results for NO_*x*_ were found in another study comparing cohorts from Italy (93 μg/m^3^), again with higher air pollution which was associated with lower average blood methylation across the Illumina 450K platform, and the Netherlands (30 μg/m^3^) where air pollution concentrations were intermediate (between the high of Italy and the low of Sweden) which did not reach significance [30, 62]. These results may indicate that the effects of NO_*x*_ on DNAm are concentration-dependent and suggest a possible threshold that lies somewhere between 7 and 93 μg/m^3^. However, it is not possible to say with certainty that effects were mediated by NO_*x*_, as in natural setting volunteers are likely to be exposed to a complex mixture of pollutants which may be produced by the same sources or otherwise correlated.

#### Additional sources of DNA methylation variability

Interestingly, global effects on DNAm may also be influenced by genomic context, as a negative correlation between NO_2_ exposure and global 450K blood DNAm was found across regions of low CpG density but not in CpG islands [30]. However, conversely, a positive correlation of DNAm in gene promoter regions with higher PM_10_ (47 μg/m^3^) concentrations was noted in a cohort from Italy. In the Netherlands cohort, with lower PM_10_ levels (25 μg/m^3^), air pollution was again associated with a global decrease in 450K DNAm, except on CpG islands, shores, and in gene promoter regions [30]. Another study found a significant association between personal PM_2.5_ exposure and DNAm at 13 CpGs, the majority of which were located in gene bodies, while seven were found in the open sea (Additional file 2: Table S2) [63]. These results may indicate that, in addition to there possibly being different thresholds at which particular air pollutants modulate global DNAm, there may also be concentration-dependent effects on DNAm at locations relative to CpG islands or gene promoters.

Differences between cohorts may alternatively be explained by variation in individuals over time. A recent study evaluated the link between air pollution exposures and DNAm in buccal cells over time in Leuven, Belgium [64]. They found that within-volunteer variation in DNAm and DNA hydroxymethylation over time was 16 and nine times higher, respectively, than between-volunteer variation. Exposure to PM_2.5_ and PM_10_ was associated with a decrease in both DNAm and DNA hydroxymethylation, with the largest effects found using a 7-day average exposure window. For every 5-μg/m^3^ increase in PM_2.5_ (relative to an average concentration ~ 20 μg/m^3^), there was a − 0.53% and − 0.015% decrease in the global DNAm and DNA hydroxymethylation, respectively. Although this study used highly accurate mass spectrometry analysis, the small size of the changes makes the biological relevance of the variability seen within and between volunteers questionable. Nevertheless, while DNAm generally constitutes a more stable biomarker than mRNA or protein expression, representing a somewhat longer time window in terms of biological responses, these results indicate that there may still be significant variability within volunteers over time that could result in false-positive or negative results [57, 64]. This variability may contribute to the finding that there is no overlap in the CpGs described in the manuscripts reviewed (Additional file 2: Table S2). This variability may reflect the importance of immune system pathways, which may show greater DNAm volatility, in the response to air pollution exposure, and this may contribute to disease susceptibility differences between individuals over time [65].

## Association of DNA methylation with lung disease-relevant outcomes

In addition to affecting gene expression, DNAm changes may be associated with lung disease development and exacerbation, our area of research interest. A study investigating the effects of air pollution exposure on lung function identified a significant association of NO_2_ exposure with blood DNAm and with lung function (FVC and FEV_1_/FVC) that was borderline significant (Table 1) [66]. However, findings for NO_2_ with DNAm were not replicated in two independent cohorts described in the same paper [66]. Nevertheless, mediation analysis suggested that one and two CpG sites mediated a significant association of NO_2_ with FVC and FEV_1_/FVC, respectively (Additional file 2: Table S2). These results indicate that DNAm modifications may act, at least in part, as an intermediary between NO_2_ exposure and lung function changes (NO_2_ → DNAm → lung function).

A similar analysis of volunteers in Boston showed that 28-day average exposures to BC, CO, PM_2.5_, or NO_2_ resulted in significantly decreased lung function, as measured by FEV_1_ and FVC, with this association modified by blood DNAm of the glucocorticoid receptor (*NR3C1*) and *IL6* [29]. However, no effects on spirometry were found in association with higher LINE1 or Alu DNAm. These results suggest that DNAm at crucial sites, rather than global status, may be associated with lung function. In evaluating specific chronic obstructive pulmonary disease (COPD) phenotypes, effects of BC, PM_2.5_, and NO_2_ exposure on FVC and O_3_ on FEV_1_ were greater in volunteers with emphysema, while those with chronic bronchitis had lower FEV_1_ values only in response to PM_2.5_ exposure [29]. A similar study in asthmatic and atopic volunteers investigating the effects of mono- and co-exposures to diesel exhaust and allergen showed that exposure order might contribute to the magnitude of the response [39]. A total of seven CpG sites were modulated by allergen, DE, or co-exposure in bronchial brushings at 48 h. However, when the same lung tissue was exposed to an allergen and then to the allergen and DE a month later, 548 CpG sites were modulated [39]. These results suggest that exposure to an allergen may prime the lungs for responsiveness, which results in more significant effects on subsequent co-exposure to air pollution and an allergen. However, the mechanisms underlying such effects at the level of DNAm are unclear.

## Mechanisms underlying the effects of air pollution on DNA methylation

The papers described above highlight the fact that air pollution exposure is associated with changes in DNAm from the earliest stages of development onwards that may contribute to disease development (Table 1). However, we do not fully understand how changes in DNAm occur. In particular, we do not know to what extent systemic effects of air pollution exposure are mediated by PM translocating from the lungs into the blood rather than, as is generally predominantly thought, mediated through the movement of cytokines from the lung into circulation [46]. Regardless, oxidative stress induced by ROS is believed central to the downstream effects of inhaled air pollution (Fig. 1). Oxidative species may also reduce the expression of methionine adenosyltransferase 1A (MAT1A) and the efficiency of the one-carbon metabolism pathway leading to a scarcity of the methyl donor SAMe needed to establish and maintain DNAm (Fig. 1) [33, 34]. Air pollution exposure can also reduce DNMT-1α expression, potentially enhancing passive dilution [18, 33]. Such effects may, over time on balance, increase the number of unmethylated cytosine sites relative to those methylated, following air pollution exposure. A decrease in 5-mC could also be mediated by enhanced conversion of 5-mC to 5-hmC, with subsequent conversion to 5-fC or 5-caC followed by excision-repair (Fig. 1).

Conversely, DNAm of the TET promoter may be enhanced by exposures, potentially resulting in decreased gene and protein expression [50]. Reduced TET protein expression and activity may favor the maintenance of 5-mC levels and, over time, promote increased methylation levels. These different mechanisms raise the suspicion that TRAP disrupts the balance between effects mediating higher DNAm and removal of methyl groups (Fig. 1). For example, changes in the overall expression of enzyme families, such as DNMT and TET, alongside processes such as oxidation and passive dilution of DNAm may lead to global hypomethylation [13, 18, 19]. It is certainly conceivable that TRAP induces more “targeted” effects on specific sites, through changes in the recruitment of transcription factors and histones that attract enzymes to mediate DNAm or demethylation, but the current evidence is too immature to portray any such targeting with sufficient consistency [13, 19].

### Intervention on air pollution-related DNA methylation

Clues to the mechanisms underlying air pollution-induced DNAm changes may come from studies testing possible interventions, as a number of papers have indicated that the harmful effects of air pollution exposure can be reduced, though not eliminated [67–69]. Limited evidence suggests benefits of exercise and supplementation with antioxidants, vitamins, and carotenoids in reducing the effects of air pollution, but replication and large-scale validation are critically needed [67, 68]. A recent controlled human exposure experiment tested B vitamin supplementation (B6, B12, and folic acid) to reduce the effects of air pollution exposure on DNAm in CD4^+^ Th cells (Table 1) [69]. PM_2.5_ exposure caused changes in DNAm that were attenuated (28–76% in the 10 most significant CpGs) after B vitamin supplementation. B vitamins may maintain DNAm levels by supplying methyl groups through the one-carbon cycle, suggesting that this may be mechanistically important (Fig. 1). Improved studies may enable a greater understanding of the mechanisms underlying the effects of air pollution exposure on DNAm.

## Common limitations of the studies to date

There are some important limiting factors in many of the papers described, including small sample sizes, low numbers of air pollution monitoring sites, variability in analysis approaches, and low reproducibility between cohorts (Table 1) [28]. Concern over the reproducibility of results is supported by an analysis of the CpGs in Additional file 2: Table S2, extracted from the main text of the papers reviewed. Surprisingly, no duplicate CpG sites were identified between any of the papers reviewed. While we do not know the reason(s) behind this lack of duplication, we can speculate as to a number of possibilities that appear linked to limitations of the papers reviewed. These range from variability in the environments of different studies, through differences in data analysis techniques to intrinsic stochasticity in DNA demethylation changes following air pollution exposure.

### Heterogeneity between studies

In the majority of studies, exposures were inferred using LUR models based on data from fixed sensors frequently a kilometer or more from volunteer’s homes [31, 57, 64]. Furthermore, LUR models do not always take into account localized exposures in areas where people spend time away from home during the day. Depending on the local source of air pollution, there are likely to be differences in the mixture of pollutants to which individuals are exposed [28]. Some studies employ measurement or statistical adjustment for different components of for example PM_2.5_, but the attribution of DNAm changes to particular elements remains challenging [70], possibly excepting single-component controlled human exposure studies [71]. A further limitation is that in most studies only a handful of specific pollutants were measured and the effects may actually be mediated by unmeasured air pollutants from the same source or whose concentrations correlate with the measured factors [8, 28]. Differences may be compounded if multiple pollutants interact, as has been shown for TRAP and allergens, depending on whether these combinations result in negative, additive, or synergistic effects on DNAm [7]. Emerging data also indicate that air pollution concentrations can vary dramatically over small areas due to traffic patterns, trees, point emission sources, and terrain, which may lead to considerable inaccuracies over shorter time windows [8, 72]. As averaging time periods evaluated in studies get longer, many spikes or troughs in air pollution concentration may be smoothed out, but controlling for other factors, such as seasonality, may become more important [28, 73]. Differences in timing between exposure and analysis may also contribute to a lack of duplication, as there may be time-dependent changes in DNAm patterns in response to air pollutants.

Additional variability may be introduced by individual characteristics, including genetic background, diet, and smoking habits, although many studies try to control for such factors [57]. Nevertheless, a previous study of the effects of diet on DNAm found no significant effects of a typical western diet, as would be expected to be consumed by the majority of participants in the studies evaluated, though they did find effects of a diet high in vegetables and fruit [74]. To the extent that an observational study assessed regional air pollution that correlated substantially with local methylation-altering foods, there could be confounding of the relationship between air pollution and DNAm endpoints.

### Variability in analytical approaches

A further technical limitation that may affect analysis is that most studies used bisulphite conversion and DNAm analysis without additional steps to isolate 5-hmC modification, and results, therefore, represent both base modifications, which may have different effects on transcription [20]. The variability inherent in sampling from small cohorts may also be exacerbated by differences in preprocessing and normalization algorithms used between studies, reflecting the lack of a standard protocol for analysis of results from the majority of DNAm analysis platforms [25, 75–77]. This lack of standardization is also often present in choices regarding cutoff values, including those for outlier samples and the array signal considered to be above background. Additionally, the majority of studies we evaluated did not report performing cell type correction (Additional file 1: Table S1), based on either cell counts or estimated via algorithmic techniques from DNAm data [78], which may mean that results are biased as air pollution exposure can modulate cell type proportions [56, 79–81]. Cell type proportions have been shown to be modulated following air pollution exposure, both directly by analysis of differential cell counts [56, 79, 81] and by indirect determination using DNAm data and Houseman or Horvath’s algorithmic approaches [80]. Differences in the statistical methods used for data analysis (e.g., linear vs linear mixed-effects models), the post-tests used for multiple testing adjustment, and the choice of significance thresholds may also contribute to false-positive and negative results, hindering study replication [43].

### Targeting of DNA methylation changes

The data reviewed suggest that in general, air pollution exposure mediates a decrease in DNAm across the genome, though methylation at some sites may be increased (Additional file 1: Table S1). Data from the use of drugs, such as low doses of decitabine (5-aza-2'-deoxycytidine) that reduce the activity and number of DNMTs, show that treatment also leads to global hypomethylation following DNA replication [82]. This occurs via an inability of DNMTs to replicate the methylation status of sites on the DNA template strand to the daughter strand, leading to dilution of DNAm. Similar untargeted effects may underly the changes induced following air pollution exposure through decreased expression and activity of DNMTs (Fig. 1) [13, 19, 83]. Alternatively, decreased MAT1A or one-carbon cycle activity may mediate a shortage of SAMe, the methyl donor, leading to global decreases in average DNAm. Stochastic or untargeted effects of air pollution on DNAm would make replication rare in global analyses (especially for methyl-seq or on the 450K/850K platforms, given the large number of sites).

The mechanisms underlying potential increases in DNAm at specific sites are less well understood [13, 19]. However, initial evidence suggests that DNMTs may use protein domains to bind to DNA in a sequence-independent manner or may be recruited to DNA sites through interactions with other proteins, including transcription factors and HDAC1, or by miRNAs. The specificity of increased DNAm following air pollution exposures and any mechanisms underlying such effects remain to be fully clarified.

### Unresolved questions regarding the effect of air pollution on DNA methylation

The previous sections suggest a range of foci in which uncertainty remains to be resolved, and other deficiencies in the literature are worth noting here [83]. For example, the extent to which exposures during specific developmental stages are most influential, relative to effects observed in relation to accumulated changes over prolonged periods, may have profound implications. Also, worth interrogation is whether or not there is a threshold concentration (or time-concentration product) of pollution that must be exceeded to be sufficient for a demonstrable health effect. Whatever the observed impact on DNAm, it would be valuable to know which particular elements of the air pollution mixture most potently drive such effects. It is also unclear whether changes in DNAm are due to global perturbations of enzymes such as TET or, if instead, effects are predominantly targeted to individual genes (Fig. 1 and Table 1) [19].

A further crucial unresolved question in environmental epigenetics is how changes in DNAm observed with acute exposures relate to the development of morbidity years later [84]. Typically, chronic exposures are assigned based on an average or other summarizing metrics, which inevitably reflects a series of shorter-term exposures that can be presumed to act cumulatively. In some, but certainly not all cases, changes in DNAm that correlate with long-term exposures are also altered by short-term exposure; a noteworthy example in the context of TRAP was recently exhibited [85], but few of such comparative attempts have been documented in humans. In a fish model comparing acute and chronic stress, 27% of the acute stress-induced changes in DNAm were also seen in response to chronic stress [86]. However, DNAm is reversible [87], and a controlled human study [55] demonstrated that circulating changes in DNAm observed within 30 h of acute exposure to TRAP alone did not carry over to a re-examination weeks later. A similar phenomenon was observed more recently in mice [88]. Since DNAm changes associated with TRAP may be at least in part remediable by acutely targeted interventions, our understanding of the temporal dynamics linking TRAP to longstanding and functionally relevant DNAm requires more investigation.

## Conclusions

DNAm is both static and dynamic, with cell-type specific patterns maintained throughout life, and environmental exposures may mediate alterations in both gene-specific and average DNAm [33, 61]. Air pollution exposure is associated with changes in DNAm across the life course, from early effects during pregnancy through to old age. Often effects on DNAm in adulthood seem most apparent in association with longer periods of exposure, suggesting that changes take time or need to accumulate, but data for temporal resolution therein remains poorly developed, and it is possible that short-term peaks have long-term effects [39]. It remains unclear if effects are accelerated by early life air pollution exposure or whether disease development simply reflects cumulative exposures. The specific mechanisms through which air pollution modulates DNAm and ultimately causes disease are poorly described. An improved understanding of the mechanisms underlying the effects of air pollution exposure may allow more targeted preventative and remedial strategies.

As we better understand how changes in DNAm are associated with effects on health, we will better differentiate between causal associations, confounding in which another factor is primarily causative of change in DNAm and downstream morbidity, or even reverse causation, in which disease processes alter DNAm (Byrne and Drake 2019). Ultimately improved knowledge of how air pollution exposures lead to changes in DNAm and thus contribute to the risk of developing lung conditions, cardiovascular disease, and mental health disorders may enable preventative strategies to minimize the development of these chronic conditions.

## Additional files

Additional file 1: **Table S1.** Enhanced detail on the studies reviewed. This table describes details of the studies described in this review, including the first author’s surname, study location, age and number of volunteers, concentrations of various air pollutants, sample types, and study details. NA, not applicable; ND, not determined; NR, not reported; BC, black carbon; CO, carbon monoxide; LINE, long interspersed nuclear element; LUR, land use regression; NO, nitric oxide; NO_2_, nitrogen dioxide; NO_*x*_, nitrogen oxides; O_3_, ozone; PM, particulate matter; PM_2.5_, particulate matter smaller than 2.5 μm; PM_10_, particulate matter smaller than 10 μm; PAH, polycyclic aromatic hydrocarbon. (XLSX 21 kb)

Additional file 2: **Table S2.** List of top air pollution-associated CpG site numbers. The main text of the papers highlighted in Table 1 was checked for lists of specific cg sites. All the cg sites were collated and ranked according to the cg number. Annotation details for the CpGs, including chromosome and location, strand, relation to CpG islands, gene symbol, and relation to genes, were merged from Illumina EPIC annotation data produced by the Kasper D. Hansen lab (www.hansenlab.org). TSS, transcription start site; UTR, untranslated region. (XLSX 21 kb)

## Data Availability

All data reviewed and described is either included in this manuscript or available online in the relevant publications.

## Abbreviations

5-caC: 5-Carboxycyotosine
5-fC: 5-Formylcytosine
5-hmC: 5-Hydroxymethylcytosine
5-mC: 5-Methylcytosine
AP-1: Activator protein-1
BAL: Bronchioalveolar lavage
BC: Black carbon
CD: Cluster of differentiation
CO: Carbon monoxide
COPD: Chronic obstructive pulmonary disease
DALYs: Disability-adjusted life years
DE: Diesel exhaust
DEP: Diesel exhaust particulate
DNA: Deoxyribonucleic acid
DNAm: DNA methylation
DNMT: DNA methyltransferase
F3: Coagulation factor III tissue factor
FEF_25–75_: Forced expiratory flow at 25–75% of the pulmonary volume
FEV_1_: Forced expiratory volume in 1 second
FOXP3: Forkhead box P3
FVC: Forced vital capacity
H_2_O_2_: Hydrogen peroxide
HLA-DRA: HLA class II histocompatibility antigen, DR alpha chain
HSD11B: Hydroxysteroid 11-beta dehydrogenase
ICAM: Intercellular adhesion molecule
IFN: Interferon
IL: Interleukin
IUGR: Intrauterine growth restriction
LINE: Long interspersed nuclear element
LUR: Land use regression
MAPK: Mitogen-activated protein kinase
MAT1A: Methionine adenosyltransferase 1A
miRNA: MicroRNA
NF-κB: Nuclear factor-kappa
NO: Nitric oxide
NO_2_: Nitrogen dioxide
NO_*x*_: Nitrogen oxides
O_3_: Ozone
PAH: Polycyclic aromatic hydrocarbon
PM: Particulate matter
PM_0.1_: Ultrafine particulate matter
PM_10_: Particulate matter smaller than 10 μm
PM_2.5_: Particulate matter smaller than 2.5 μm
ROS: Reactive oxygen species
SAMe: S-Adenosyl methionine
SLC25A28: Solute carrier family 25 member 28
SO_2_: Sulfur dioxide
TDG: Thymine DNA glycosylase
TET: Ten-eleven translocation methylcytosine dioxygenase
TPO: Thyroid peroxidase
TRAP: Traffic-related air pollution
WHO: World Health Organization
